# Powdery Mildew Resistance in Tomato by Impairment of *SlPMR4* and *SlDMR1*

**DOI:** 10.1371/journal.pone.0067467

**Published:** 2013-06-20

**Authors:** Robin P. Huibers, Annelies E. H. M. Loonen, Dongli Gao, Guido Van den Ackerveken, Richard G. F. Visser, Yuling Bai

**Affiliations:** 1 Laboratory of Plant Breeding, Wageningen University and Research Centre, Wageningen, The Netherlands; 2 Plant-Microbe Interactions, Department of Biology, Utrecht University, Utrecht, The Netherlands; Ghent University, Belgium

## Abstract

Genetic dissection of disease susceptibility in Arabidopsis to powdery and downy mildew has identified multiple susceptibility (*S*) genes whose impairment results in disease resistance. Although several of these *S*-genes have been cloned and characterized in more detail it is unknown to which degree their function in disease susceptibility is conserved among different plant species. Moreover, it is unclear whether impairment of such genes has potential in disease resistance breeding due to possible fitness costs associated with impaired alleles. Here we show that the Arabidopsis *PMR4* and *DMR1*, genes encoding a callose synthase and homoserine kinase respectively, have functional orthologs in tomato with respect to their *S*-gene function. Silencing of both genes using RNAi resulted in resistance to the tomato powdery mildew fungus *Oidium neolycopersici*. Resistance to *O. neolycopersici* by *SlDMR1* silencing was associated with severely reduced plant growth whereas *SlPMR4* silencing was not. *SlPMR4* is therefore a suitable candidate gene as target for mutagenesis to obtain alleles that can be deployed in disease resistance breeding of tomato.

## Introduction

High quality and productive crops are often susceptible to a multitude of different pathogens and pests. Disease resistant crop varieties are commonly bred by the introgression of resistance (*R*) genes derived from wild crop relatives. Such *R*-genes often mediate recognition of race or isolate-specific effector proteins of the pathogen and the subsequent activation of defense responses in the plant. However, the frequent appearance of ‘new’ pathogen races in terms of their effector protein arsenal, renders many introgressed *R*-genes quickly ineffective, initiating a new cycle of *R*-gene discovery and introgression by breeders. Disease resistance breeding approaches that could overcome this lack of durability would therefore be highly advantageous.

Disease resistance can be obtained via several means. EMS mutagenesis approaches have regularly been used in Arabidopsis for varying purposes. Researchers working with Arabidopsis have selected a plethora of mutants showing different kinds of activated defense response phenotypes, such as *cpr* mutants (constitutive expression of *PR* genes, [1]), *agd* mutants (aberrant growth and death phenotypes, [2]), *acd* mutants (accelerated cell death, [3]), *lsd* mutants (lesions simulating disease, [4]), or *dnd* mutants (defense no death, [5]). In general, these mutants show race-non-specific resistance to a broad spectrum of different pathogens. However, resistance is often associated with severe fitness costs, apparent from the small size and reduced fertility of these mutants.

Mutant screens which were performed later and aimed at dissecting the genetic basis of disease susceptibility, identified so called loss-of-susceptibility mutants showing disease resistance in absence of severe fitness costs [6]–[8]. This, together with recent insights in effector triggered susceptibility by manipulation of host factors led to a proposed new breeding strategy [9]. Instead of identifying *R*-genes in wild relatives and the subsequent introgression of these genes into elite cultivars, one could inactivate so called ‘*Susceptibility’ (S)* genes by mutagenesis. As resistance is not mediated by race-specific recognition of pathogens but by the absence of certain host genes, the *S*-gene approach is expected to be more durable. This is exemplified by powdery mildew resistant barley *mlo* lines, which have been successfully cultivated in agriculture for over 25 years [10].

*Mlo* based powdery mildew susceptibility is conserved across plant species as impairment of barley *Mlo* orthologs in Arabidopsis [11], tomato [12] and pea [13]–[14] all result in resistance to adapted powdery mildews. For other *S*-genes it is unclear to which degree functions are conserved across plant families and whether impairment of such genes have potential breeding value. Here we report the functional characterization of tomato (*Solanum lycopersicum*) orthologs of the Arabidopsis *PMR4* and *DMR1* genes with respect to their *S*-gene function. *Powdery Mildew Resistant* (*PMR*) *4* was originally identified in a mutant screen for loss of susceptibility to *Erysiphe cichoracearum* [6]. *PMR4* encodes a callose synthase required for callose deposition in papillae. Resistance in *pmr4* mutants, however, was found associated with salicylic acid accumulation rather than with the absence of callose deposition [15]. *D*owny *M*ildew *R*esistance (*DMR*) *1* was identified in a mutant screen for loss of susceptibility to *Hyaloperonospora arabidopsidis* [7]. Several different *dmr1* alleles were identified, each mediating a significant level of resistance to *H. arabidopsidis*. *DMR1* encodes a homoserine kinase catalyzing the phosphorylation of homoserine to O-phospho-homoserine. Mutant plants accumulate homoserine and exogenous application of homoserine induces resistance in wild-type plants [16]–[17]. We observed reduced growth and proliferation of *Oidium neolycopersici* (tomato powdery mildew) in Arabidopsis *dmr1* and *pmr4* mutants as well as tomato plants in which expression of *SlDMR1* and *SlPMR4* was silenced, demonstrating that these *S*-gene functions are conserved between the two plant species. Resistance to *O. neolycopersici* is associated with severe fitness cost for *SlDMR1* silencing but not for *SlPMR4* silencing, indicating the latter has potential value for powdery mildew resistance breeding in tomato.

## Materials and Methods

### Plant Growth and Cultivation

*Arabidopsis thaliana* mutants *pmr4* in Col-0 background and *dmr1-1* to *dmr1-6* in L*er eds1-2* background are described in [6], [7]. Plants were grown in plastic pots in potting soil (Horticoop, Lentse potgrond). Arabidopsis plants were grown in a growth chamber at 21°C and 19°C during the 8 h day and 16 h night periods respectively, a relative humidity of 70% and a light intensity of 100 W/m^2^. Tomato plants (*S. lycopersicum* cv Moneymaker) were grown in greenhouses at 21°C and 19°C during the 16 h day and 8 h night periods respectively. Relative humidity was around 70% and light intensity was supplemented with 100 W/m^2^ when light intensity dropped below 150 W/m^2^.

### Pathogen Inoculations

The Wageningen isolate of *Oidium neolycopersici* On-Ne [24] was maintained on tomato cv Moneymaker (MM) plants. Spore suspensions were obtained by washing heavily infected MM leaves in water. For disease assays, Arabidopsis and tomato plants of about 4 weeks old were sprayed with an inoculum of between 5–10×10^4^ and 2.5×10^4^ spores per mL respectively. Fungal growth was evaluated at 8–14 days post inoculation (dpi). A disease index (DI) was used where 0 = no sporulation; 1 = slight sporulation, but less than 5% foliar area affected; 2 = moderate sporulation, 5 to 30% foliar area affected; 3 = abundant sporulation, 30% to 60% foliar area affected; 4 = heavy sporulation, more than 60% area affected. Fungal quantification by Q-PCR was performed after 8–14 dpi (see below).

### Identification of Tomato Orthologs

The *Arabidopsis thaliana* DMR1 (GenBank accession number AEC06605.1) and PMR4 (GenBank accesssion number AEE82336.1) amino-acid sequences were used as a query in a TBLASTN program against the SGN Tomato Combined database (http://solgenomics.net/tools/blast/) or in a BLASTP program against an Arabidopsis protein database (http://www.arabidopsis.org/Blast/index.jsp) to search for homologous sequences in tomato and Arabidopsis respectively. All obtained tomato and Arabidopsis amino-acid sequences were aligned and phylogenetic trees constructed by using Phylogeny.fr [25]. Tomato sequences showing a higher level of homology with PMR4 or DMR1 compared to any other Arabidopsis sequence were considered orthologs.

### Generation of Silencing Lines

For generating the *SlPMR4* and *SlDMR1* silencing constructs we ordered pUC57 clones containing a DNA fragment identical to the first 101 bp of the predicted coding sequence of *Solyc07g053980* (SlPMR4_h1) or a fragment identical to the first 140 bp of the predicted 3′ UTR of *Solyc04g008760* (SlDMR1) flanked by attL sites from the Genscript (USA) company. Sequences are provided in Figure S1 in File S1. DNA fragments present in pUC57 were recombined into pHellsgate 8 [26] using the LR clonase enzyme mix from Invitrogen and transformed to chemical competent *E. coli* TOP 10 cells (Invitrogen). Constructs were subsequently extracted using the Plasmid extracting kit from Qiagen, sequenced to confirm the presence of the intended inserts and transformed to Agrobacterium strain AGL1+virG. For tomato transformation Moneymaker (MM) seeds were sterilized by incubation in 1% NaOCl for 20 minutes. Afterwards seeds were rinsed with water and sown on GEM medium (2.2 g MS salts/L, 10 g sucrose/L and 8 g Daishin agar/L; pH 5.8). Cotyledons from emerging plants were cut in several pieces and incubated for 10–15 minutes in an Agrobacterium suspension carrying the silencing constructs (OD600 of 0.125) in MSO (4.3 g MS salts/L, 0.4 mg thiamine/L, 100 mg Myo-inositol/L and 30 g sucrose/L; pH 5.6) plus 200 µM acetosyringone. Then the cotyledon pieces were blotted on sterile filter paper and placed with abaxial side down on GCF10 medium (4.3 g MS salts/L, 108.7 mg Vitamins Nitsch/L, 30 g sucrose/L, 8 g agar/L, 1.5 mg Zeatine riboside/L and 0.2 mg IAA/L; pH 5.8) plus 200 µM acetosyringone. This was then incubated in the dark for 48 hours at 25°C before transferring the explants to plates containing GCF10 medium plus 300 mg/L timentin and 100 mg/L kanamycin and subsequent incubation at 25°C in the light. After 3 weeks, the medium was refreshed. After 6–8 weeks emerging calli were excised from explants and transferred to GCF11 medium (4.3 g MS salts/L, 108.7 mg Vitamins Nitsch/L, 30 g sucrose/L, 8 g agar/L and 1.9 mg Zeatine riboside/L; pH 5.8) plus 300 mg/L timentin and 100 mg/L kanamycin. Emerging shoots were transferred to MS30B5 medium (4.3 g MS salts/L, 112 mg Vitamin B5/L, 30 g sucrose/L and 8 g agar/L; pH 5.8) plus 100 mg/L kanamycin. When proper roots had developed transformants were brought to the greenhouse. They were transferred to plastic pots containing potting soil, and kept under a plastic cover for a few days to achieve higher humidity. After adaptation the plastic cover was removed. In total, eight *RNAi::SlPMR4* and twelve *RNAi::SlDMR1* primary transformants (T1) were grown in the greenhouse. After gene expression analysis (see below) for each silencing construct three T1 plants that showed the highest level of silencing were selected, and allowed to set seed (T2). For *SlPMR4*-silenced plants, from each T1 plant 16–20 T2 progeny was grown in the greenhouse, together with 19 MM plants as control, and used in a disease assay.

### Nucleic Acid Extraction and Q-PCR

To determine presence of the silencing contruct DNA was isolated from leaf material using the HotSHOT method [27]. A PCR was performed using NPTII-specific primers NPT3 (TCGGCTATGACTGGGCACAACAGA) and NPT4 (AAGAAGGCGATAGAAGGCGATGCG) [28]. For gene expression analysis, 8–14 days after inoculation of tomato plants with *O. neolycopersici* the 3^rd^ or 4^th^ leaf was collected from each T1 or T2 transformant or MM control plant. Total RNA was extracted using the MagMAX-96 total RNA Isolation kit (Ambion) or the RNeasy kit from Qiagen (Germany). RNA was treated with RNAse-free DNase (Qiagen). The quantity of RNA was measured using a spectrophotometer and 250 ng-1 µg was used to synthesize cDNA using the iScript cDNA synthesis kit (Bio-Rad). Relative transcript levels were determined in two technical replicates using the iQ SYBR Green supermix (Bio-Rad) and the CFX96 Real-Time system (Bio-Rad). PCR was performed with 10 ng cDNA using an annealing temperature of 60°C, and 40 cycles. To check for DNA contamination samples that had not been treated with reverse transcriptase were included as a control. Tomato *EF1α* (Solyc06g005060) transcript levels were used for normalisation by the ΔΔCt method. Primers used for determining relative transcript levels were: Fw- EF1α- ATTGGAAACGGATATGCCCCT; Rv-EF1α-TCCTTACCTGAACGCCTGTCA; Fw-*SlPMR4*-GCCGGCGGCGAGACAAGTTT; Rv-*SlPMR4*-CAGCGCCAGCCAGTCAAGCA; Fw-*SlDMR1*-TGGGGAGAGAATGGTGGAGGCG; Rv-*SlDMR1*- ACTCGTTTGGAATTGAGGCATAGTTGA. Primers to determine expression of the NPTII gene were: Fw-NPTII-ACTGGGCACAACAGACAATC and Rv-NPTII-TCGTCCTGCAGTTCATTCAG.

Disease severity was measured by Q-PCR quantification of *O. neolycopersici* biomass using the same samples as used for gene expression analysis. PCR was performed using primer pair Fw-On-CGCCAAAGACCTAACCAAAA and Rv-On-AGCCAAGAGATCCGTTGTTG, designed on ITS sequences specific to *O. neolycopersici* (GenBank accession number EU047564). The Fw- EF1α and Rv-EF1α primer pair was used as reference to normalize the plant DNA proportion by the ΔΔCt method.

### Exogenous Application of Amino-acid Solutions

Amino acids L-homoserine, L-isoleucine, L-leucine, L-threonine and L-valine were obtained from Sigma-Aldrich. 5 mM solutions were sprayed onto leaves of four-weeks old tomato plants. The next day plants were inoculated with *O. neolycopersici*. Three days after inoculation the plants were sprayed again with the respective amino acid solution.

### Statistical Analysis

For comparisons of means analysis of variance was performed. When significant differences were found (p<0.05) a Tukey post hoc test was carried out to detect which means were different.

## Results

### Arabidopsis pmr4 Mutants are Resistant to O. neolycopersici

**Figure 1:**
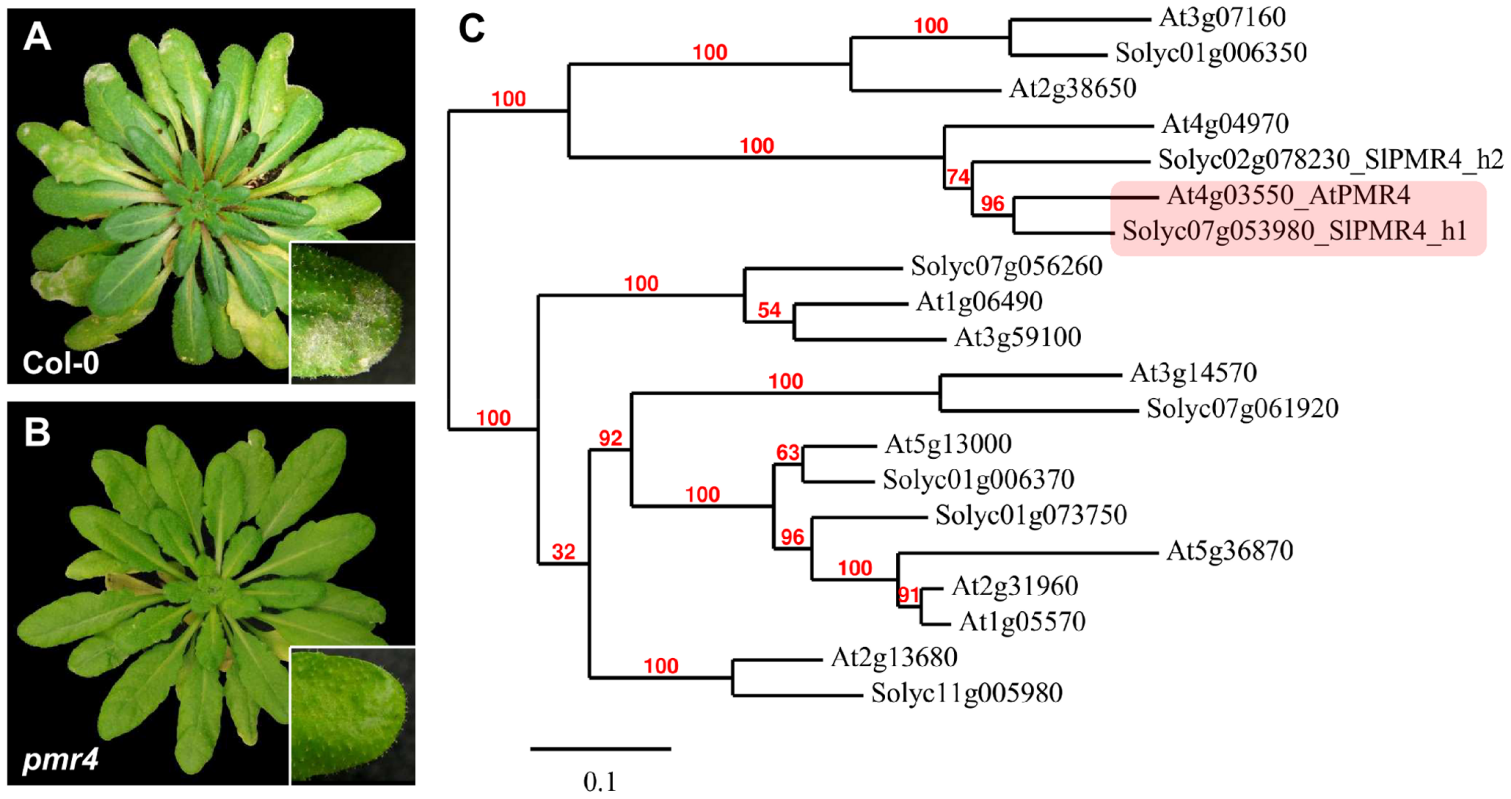
Impairment of *PMR4* in Arabidopsis results in resistance to tomato powdery mildew *Oidium neolycopersici*. (**A and B**), Col-0 and *pmr4* mutant plants photographed 14 days post inoculation with *Oidium neolycopersici*. Fungal sporulation is visible as whitish powder on multiple leaves of Col-0 but not on *pmr4* mutants, showing that Arabidopsis *PMR4* is a susceptibility (*S*) gene for *O. neolycopersici*. (**C**), Phylogenetic tree of Arabidopsis PMR4 family members plus tomato PMR4 family orthologs. The protein SlPMR4_h1 encoded by the tomato gene *Solyc07g053980* is considered to be the tomato ortholog of AtPMR4.

Previously *PMR4* was identified as a locus conferring susceptibility to the powdery mildew *E. cichoracearum* in Arabidopsis [6], [15]. As Arabidopsis can be a host for the tomato powdery mildew *O. neolycopersici* [18] we assessed the possible requirement of *PMR4* for this pathogen. Inoculated *pmr4* mutants showed reduced levels of fungal sporulation as compared to Col-0 wild-type plants (Figure 1A and B), indicating that Arabidopsis *PMR4* is a functional *S*-gene for tomato powdery mildew.

### Powdery Mildew Resistance by Impairment of PMR4 is Conserved between Arabidopsis and Tomato in Absence of Severe Fitness Costs

To explore the possible conservation of *PMR4* among different plant species for susceptibility to powdery mildews, putative tomato orthologs of Arabidopsis *PMR4* were identified by searching the SOL Genomics Network (SGN) database for similar sequences. Two genes annotated as *Solyc07g053980* (*SlPMR4_h1*) and *Solyc02g078230* (*SlPMR4_h2*) were found, encoding proteins having higher sequence identity at the amino acid level with *At*PMR4 (76% and 67% respectively), than *At*PMR4 with any of its Arabidopsis family members. Phylogeny.fr [25] was used to construct a phylogenetic tree of the Arabidopsis and tomato PMR4 family members (Figure 1C). As *Solyc07g053980* (*SlPMR4_h1*) has the highest level of homology with *PMR4* it was considered the tomato gene most likely to have similar function, and hereafter is referred to as *SlPMR4*.

**Figure 2:**
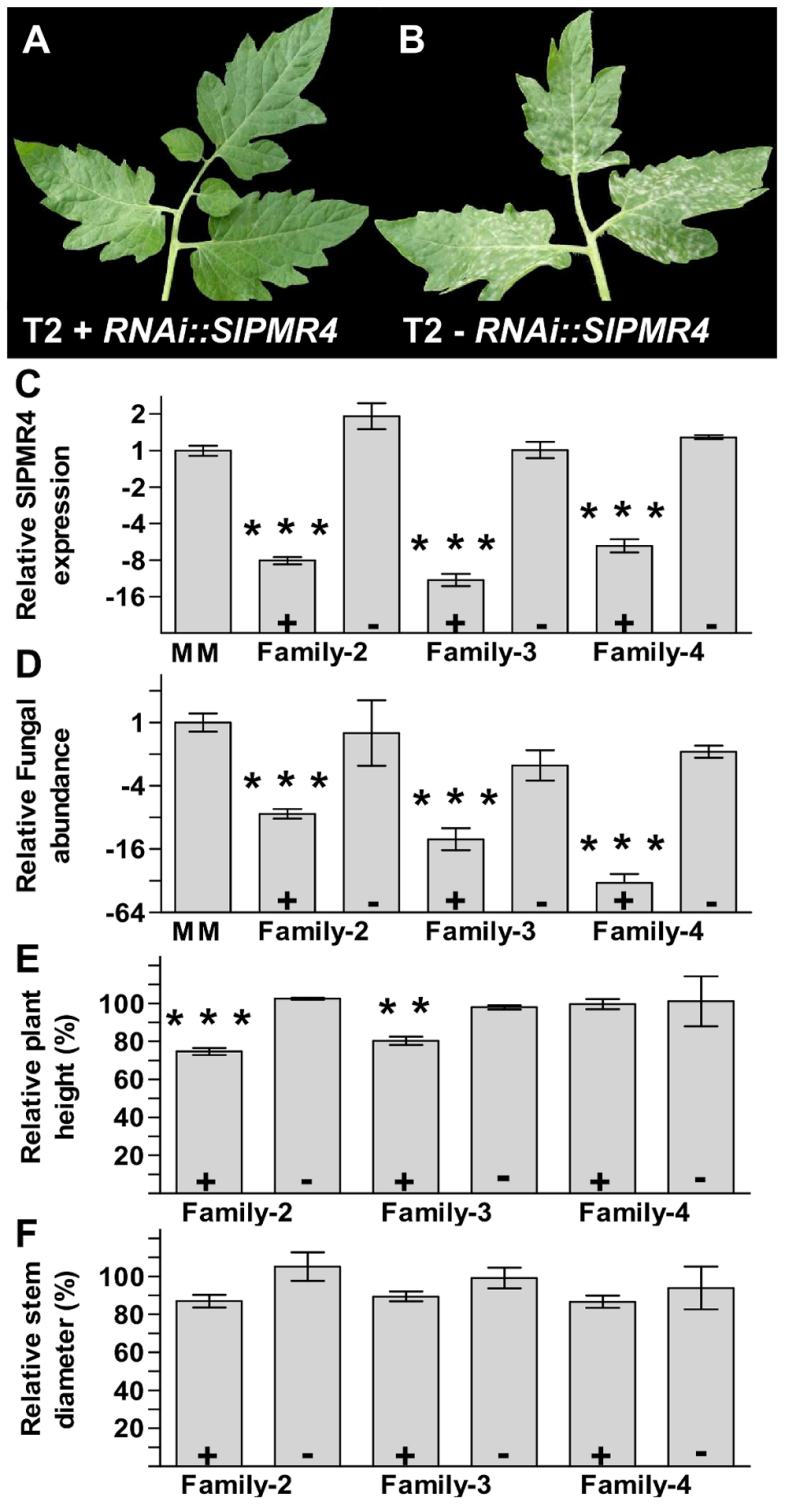
Powdery mildew resistance by impairment of *PMR4* is conserved between Arabidopsis and tomato. (**A and B**), Leaf of T2 plant 4–5 (A) carrying a *RNAi::SlPMR4* silencing construct (+) and of T2 plant 4–4 (B) without silencing construct (−) at the age of 5 weeks and 8 days post *O. neolycopersici* inoculation. (**C**), Relative *SlPMR4* transcript levels in untransformed Moneymaker (MM) plants and progeny of 3 independent T1 plants transformed with a silencing construct specifically targeting *SlPMR4* (family 2, 3 and 4). T2 progenies harbouring a silencing construct (RNAi::*SlPMR4* (+)) showed significantly lower transcript levels compared to untransformed Moneymaker plants and progenies not harbouring a silencing construct (−) as indicated by asterisks. (**D**), Quantification of fungal growth of lines mentioned in (C). *SlPMR4* silenced plants show significantly less fungal growth compared to non-silenced plants as indicated by asterisks, demonstrating that *SlPMR4* is a functional ortholog of *PMR4*. (**E and F**), Relative plant height and stem diameter of 12 week old plants respectively. A slight reduction (although significant as indicated by asterisks) in plant height was observed for progenies carrying a *RNAi::SlPMR4* construct of families 2 and 3, but not 4. Stem diameters were somewhat lower among T1 progenies of all 3 families, but differences were not statistically significant. For **C**, **D**, **E** and **F**, data indicate the mean of 3 or more biological replicates with error bars representing the standard error. Number of asterisks indicate degree of significance (**p<0.01; ***p<0.001).

To assess the potential involvement of *SlPMR4* in powdery mildew susceptibility, tomato Moneymaker plants were transformed with a silencing construct specifically targeting transcripts of this gene *Solyc07g053980* (*SlPMR4_h1*). Results on gene expression showed that with this construct no cross-silencing of *Solyc02g078230* (*SlPMR4_h2*) occurred (Figure S2 and Table S1 in File S1). The level of silencing in independent T1 plants was determined and 3 plants showing >5 fold silencing were allowed to set seeds (hereafter referred to as T2 family 2, 3 and 4). Segregation within each T2 family (3 families in total) for the presence or absence of the silencing construct correlated with the presence of relatively low or high levels of *SlPMR4* transcripts respectively (Figure 2C and Figure S2 in File S1). T2 families were inoculated with *O. neolycopersici* and their level of susceptibility determined by quantification of the relative ratio between fungal and plant DNA at 8 dpi. Fungal growth was significantly less among silenced plants compared to non-silenced plants demonstrating *SlPMR4* is a functional *S*-gene ortholog of Arabidopsis *PMR4* (Figure 2D). Resistant plants did not show any apparent reduction in size or altered leaf morphology as compared to susceptible progeny at the age of five weeks when inoculations were performed (Figure 2A and B). However, T2 progeny of family 2 and 3 harbouring a silencing construct showed slightly reduced growth after 12 weeks in the greenhouse compared to plants not harbouring a silencing construct (Figure 2E). In contrast, no significant difference in plant height was found for progeny of family 4 grown under the same conditions as progenies of families 2 and 3 (Figure 2E). Moreover, stem diameter at 12 weeks was on average reduced in the SlPMR4-silenced plants compared to the non-silenced progenies of all 3 families, although this difference was not statistically significant (Figure 2F). Together the data suggests that *O. neolycopersici* resistance by impairment of *SlPMR4* function can be achieved without severe growth reduction.

### Impairment of DMR1 is Associated with O. neolycopersici Resistance

**Figure 3:**
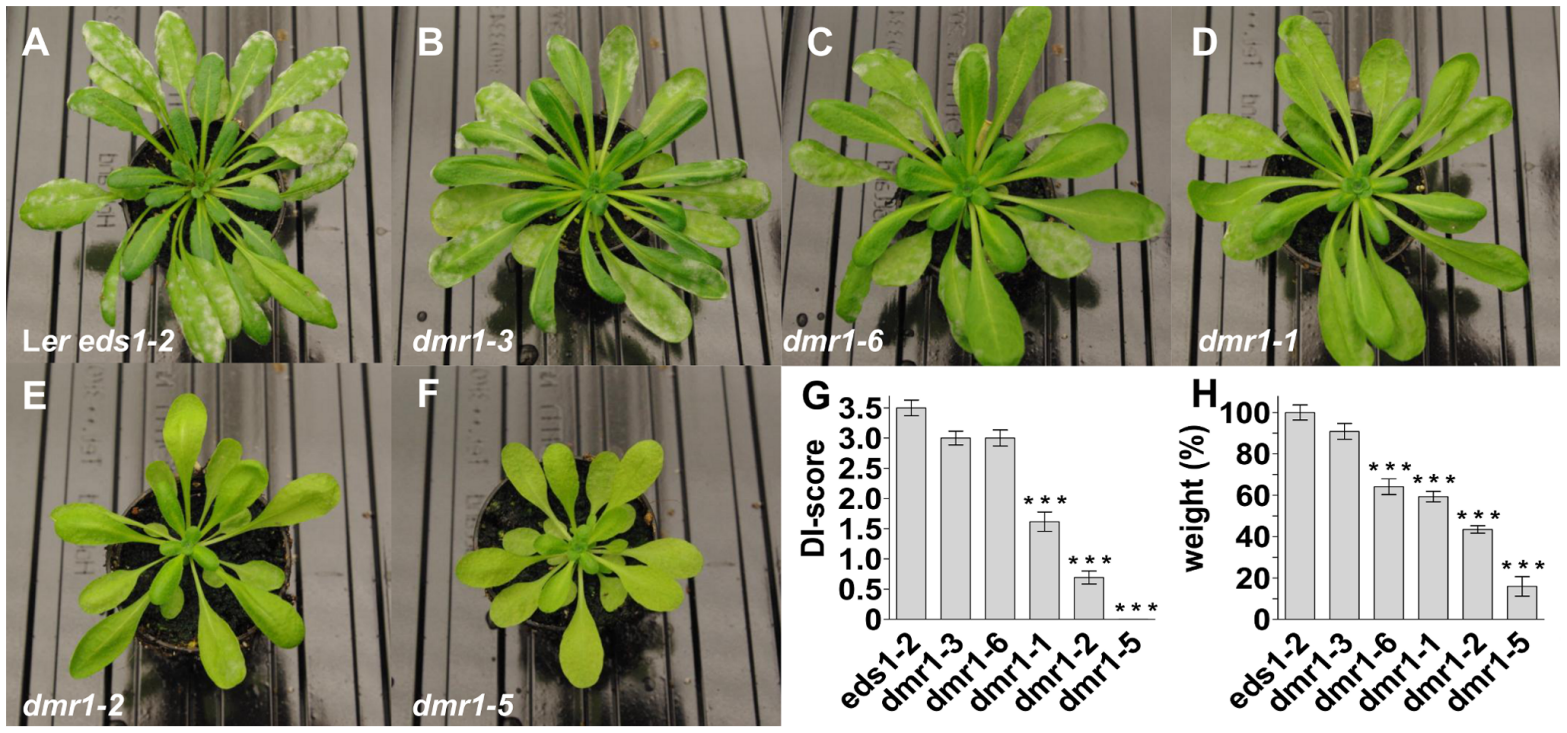
Arabidopsis *DMR1* is a S-gene for the tomato powdery mildew *Oidium neolycopersici*. (**A–F**), Photograph of L*er eds1-2* (A), *dmr1-3* (B), *dmr1-6* (C), *dmr1-1* (D), *dmr1-2* (E) and *dmr1-5* (F) at 14 days post inoculations (dpi) with *O. neolycopersici*. (**G**), Quantification of *O. neolycopersici* growth at 14 dpi by Disease Index (DI) score (see M&M). (**H**), Fresh weight of 5 weeks old L*er eds1-2* and *dmr1* mutant plants. Fresh weights are given as percentage of the parental line L*er eds1-2*. For **G** and **H**. Data indicate the mean of 3 or more biological replicates with error bars representing the standard error. Number of asterisks indicate degree of significance (***p<0.001).

Previously, Arabidopsis *dmr1* mutants were shown to be resistant to the downy mildew *H. arabidopsidis* but not to the powdery mildew *Golovinomyces orontii* or the bacterial pathogen *Pseudomonas syringae*, which suggested *DMR1* mediates susceptibility specifically to downy mildew [7]. However, we observed a significant reduction of tomato powdery mildew *O. neolycopersici* sporulation at 14 dpi on *dmr1-1*, *dmr1-2* and *dmr1-5* plants as compared to the parental line L*er*-*eds1-2* (Figure 3A–G), suggesting that *DMR1* is a functional *S*-gene for tomato powdery mildew in Arabidopsis.

All *dmr1* mutants except *dmr1-3* showed reduced growth compared to the parental line as evidenced by the lower amount of fresh weight recorded 5 weeks after sowing (Figure 3H), suggesting that impairment of *DMR1* has fitness costs. Reduction in growth was lowest in *dmr1-3* and *dmr1-6*, the same lines that did not show resistance to *O. neolycopersici*. This suggests that the weaker *dmr1* alleles do not effectively mediate resistance to tomato powdery mildew, in contrast to *H. arabidopsidis* [7].

### Pathogen Resistance by Impairment of DMR1 is Conserved between Arabidopsis and Tomato

**Figure 4:**
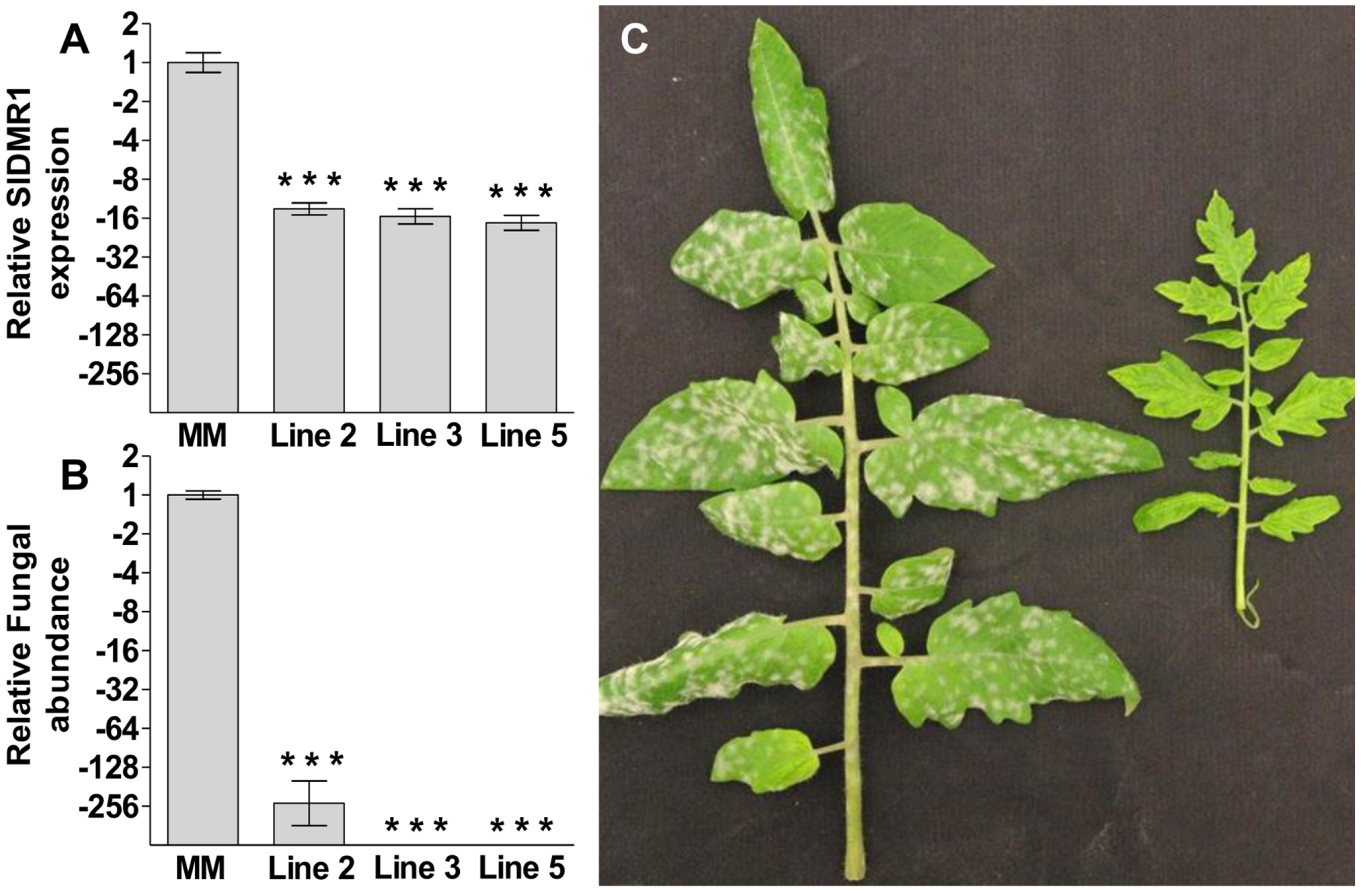
Tomato *SlDMR1* is a S-gene for the tomato powdery mildew *Oidium neolycopersici*. (**A**), relative transcript abundance of *SlDMR1* in multiple independent cuttings of untransformed Moneymaker (MM) plants or T1 transformants harbouring an RNAi::*SlDMR1* construct (Line2, 3 and 5). (**B**), Quantification of fungal growth at 8 days post inoculation (dpi). *SlDMR1* silenced plants support significantly less fungal growth compared to untransformed plants. (**C**), Leaves of an untransformed MM and *SlDMR1* silenced plant at 8 dpi. Silencing of *SlDMR1* results in reduced leaf size and yellowish colour. For **A** and **B**. Data indicate the mean of 3 or more biological replicates with error bars representing the standard error. Number of asterisks indicate degree of significance (***p<0.001).

Using the *At*DMR1 amino acid sequence as a query the SOL Genomics Network (SGN) database was searched for similar sequences. A single tomato gene annotated as *Solyc04g008760* was identified encoding a putative homoserine kinase having 71% sequence identity with *At*DMR1. A silencing construct was generated specifically targeting this gene, hereafter referred to as *SlDMR1*, and used to transform tomato MM plants. The relative transcript level of *SlDMR1* was determined in 12 independent transformants. We only obtained T1 plants showing either less than 2-fold silencing of *SlDMR1* or over 4-fold silencing. Plants showing over 4-fold silencing were of reduced stature, had light green leaves (Figure 4C) and in addition, produced no offspring. Progeny of T1 plants showing less than 2 fold silencing of *SlDMR1* did not show reduced growth of *O. neolycopersici* (data not shown). As no T2 progeny of T1 plants showing over four fold silencing could be obtained, multiple cuttings were generated for three T1 plants (Lines 2, 3, and 5) and untransformed MM plants to perform disease assays with *O. neolycopersici*. All three *SlDMR1* silenced lines (Figure 4A) showed significantly less growth of *O. neolycopersici* at 8 dpi (Figure 4B and C) as compared to untransformed MM plants demonstrating that *SlDMR1* is a functional *S*-gene in tomato.

### Homoserine Induces Powdery Mildew Resistance in Tomato

**Figure 5:**
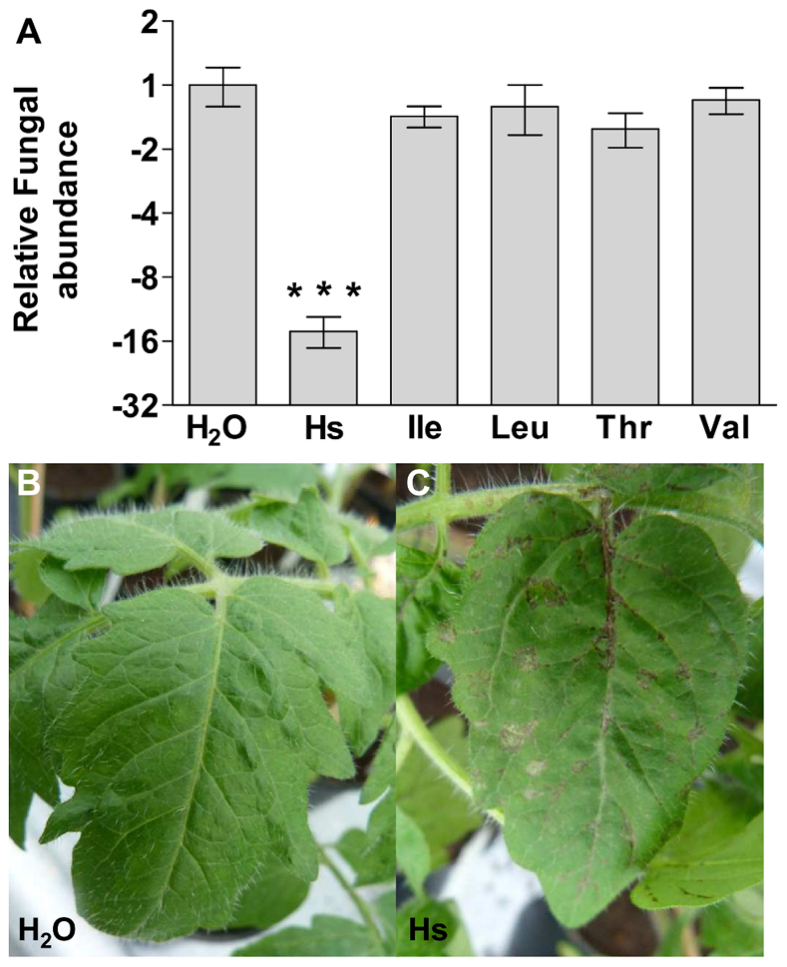
Exogenous application of L-homoserine induced *Oidium neolycopersici* resistance and necrosis in tomato. (**A**), Quantification of fungal growth, 8 days post inoculations of Moneymaker (MM) plants sprayed with different amino-acid solutions prior to pathogen inoculations. Spray application of L-homoserine reduces fungal growth significantly compared to spray application of H_2_O or isoleucine, leucine, threonine or valine. Data indicate the mean of 3 or more biological replicates with error bars representing the standard error. Number of asterisks indicate degree of significance (***p<0.001). (**B–C**), Spray application of L-homoserine induces cell death in MM plants whereas the H_2_O control does not.

Arabidopsis *dmr1* mutants accumulate homoserine [16] and exogenous application of 5 mM L-homoserine was shown to induce resistance to *H. arabidopsidis* [16]–[17]. To explore whether L-homoserine could trigger *O. neolycopersici* resistance in tomato, plants were sprayed with several amino-acid solutions prior to pathogen inoculation. Spray application of 5 mM of L-homoserine, but not of L-isoleucine, L-leucine, L-threonine or L-valine reduced infection of tomato leaves with *O. neolycopersici* (Figure 5A). In addition, L-homoserine induced necrosis in tomato leaves independent of powdery mildew infection (Figure 5B and C) suggesting (high) levels of L-homoserine are toxic to the plant.

## Discussion

The potential of knocking-down *S-*genes for durable disease resistance breeding in crops has largely been unexplored due to anticipated fitness costs of impaired *S*-gene alleles. However, identification of pathogen resistant mutants with no or minor associated fitness costs, together with recent insights in pathogen effector-triggered susceptibility by manipulation of host factors has brought (renewed) interest to this subject [9]. Here we tested if the *S*-gene function of *PMR4* and *DMR1* is conserved between Arabidopsis and tomato and explored the potential use of the tomato orthologs for breeding resistance to tomato powdery mildew.

We showed that Arabidopsis mutants harboring mutations in *PMR4* or *DMR1* are resistant to *O. neolycopersici* in addition to *E. cichoracearum* and *H. arabidopsidis* respectively, as was previously described [6]–[7]. Silencing of the tomato orthologs *SlPMR4* and *SlDMR1* resulted in *O. neolycopersici* resistance in tomato, indicating that their *S*-gene functions are conserved across plant species. Interestingly, no severe fitness costs in terms of reduced growth were found associated with *SlPMR4* silencing (Figure 2A, B, E and F) making this gene a promising target for mutagenesis to obtain suitable *Slpmr4* alleles for disease resistance breeding in tomato.

### Powdery Mildew is Less Sensitive to dmr1-mediated and Homoserine- induced Resistance than Downy Mildew

All Arabidopsis *dmr1* mutants were identified in a screen for loss of susceptibility to the oomycete *H. arabidopsidis* and were shown to be resistant to this pathogen, including *dmr1-3* [16]. No significant reduction in plant growth was observed for this mutant (Figure 3H), indicating that *H. arabidopsidis* resistance can be obtained by impairment of *DMR1* in absence of severe fitness costs. This might be different for *dmr1*-mediated resistance to the ascomycete *O. neolycopersici*, as *dmr1-3* (and *dmr1-6*) did not show a significant reduction in fungal growth (Figure 3G). Moreover, *O. neolycopersici* resistance in tomato was only observed for RNAi lines in which transcript levels were over four-fold reduced (Figure 4A,B). There was, however, a severe negative effect in the strongly silenced *SlDMR1* lines as they showed growth retardation and did not produce offspring.

Arabidopsis *dmr1* mutants accumulate homoserine and exogenous application of homoserine induces pathogen resistance [16]–[17]. Of all characterized *dmr1* alleles, *dmr1-3* encodes a homoserine kinase with the highest residual enzyme activity and homoserine accumulation was less in *dmr1-3* mutants compared to other *dmr1* mutants [16]. As *dmr1-3* is resistant to *H. arabidopsidis* but not to *O. neolycopersici* it suggests that the first is more sensitive to homoserine accumulation in the host than the latter.

### Mechanism of dmr1 and pmr4 Mediated Resistance

The mechanism by which impairment of *DMR1* mediates resistance is unclear. Experiments to test direct toxicity showed that *H. arabidopsidis* spore germination and germ tube growth is not inhibited by homoserine [16]. Impairment of *DMR1* more generally perturbs amino acid homeostasis as *dmr1* mutants show changes in their levels of the homoserine-derived amino acids threonine, isoleucine and methionine [16]. Perturbation of amino acid homeostasis was also observed in *rsp1* and *rsp2* mutants that accumulate lysine, threonine, methionine and isoleucine and are resistant to *H. arabidopsidis* but not the powdery mildew *G. orontii* [17]. In the study of Stuttmann et al. [17], exogenous application of threonine appeared to be a more potent inducer of *H. arabidopsidis* resistance than homoserine, in contrast to what was found by Van Damme et al. [16] and what we observed in tomato (Figure 5A). However, Stuttmann et al. [17] hypothesized that plants and oomycetes biosynthesize lysine via the diaminopimelate (DAP) pathway, whereas ascomycetes use the a-aminoadipate (AAA) pathway. Differences in interference of these biosynthetic pathways in the pathogen by amino acid perturbations in the host (or exogenously applied) might underlie the relative vulnerability of downy mildew (oomycete) as compared to powdery mildew (ascomycete).

There is no evidence for the activation of plant defence responses in the *dmr1* mutants or in homoserine-treated plants that are resistant to *H. arabidopsidis*. The SA-dependent defence marker gene *PR-1* was not induced in *dmr1* mutants, nor was homoserine-induced resistance impaired in a large collection of Arabidopsis defence-signalling mutants [16]. In contrast, the Arabidopsis *pmr4* mutants show constitutive activation of SA-dependent defences [15]. Resistance in *pmr4* mutants was lost in double mutants in which the SA pathway is impaired [15]. This result indicated that SA accumulation rather than lack of pathogen-induced PMR4 callose synthase activity is the cause of resistance in *pmr4* mutants to adapted powdery mildews. Recently, it was shown that overexpression of *PMR4* resulted in elevated early callose deposition, leading to complete penetration resistance to both nonadapted and adapted powdery mildews. Further, overexpression of *PMR4* did not influence SA and jasmonate-related pathways [29]. Therefore, PMR4 not only has an enzymatic function (callose synthase activity) but also plays a regulatory role (such as a negative regulator of SA pathway).

We expect that the resistance found in our *SlPMR4-*silenced tomato lines may be due to the regulatory role of the *PMR4* gene on defence-related pathways. As to the resistance shown in *SlDMR1-*silenced tomato lines, we expect that the direct toxicity of homoserine may be the cause, although we cannot exclude the possibility that the activation of plant defence responses may play a role. Further experiments are on-going to monitor the fungal growth, callose deposition and marker genes for defence pathways in *SlPMR4-* and *SlDMR1-*silenced lines. In addition, these RNAi lines are being crossed with tomato mutants defective in SA, jasmonic acid and ethylene pathways to verify whether the impairment of certain pathways compromises the resistance resulting from the disruption of *SlPMR4* or *SlDMR1.* The obtained results will clarify whether different mechanisms and/or cross-talk between the defence-related pathways are associated with the silencing of *SlPMR4* or *SlDMR1.*

### Perspective for Breeding

Making use of impaired *S*-gene alleles to obtain durable disease resistance was recently proposed as a new breeding strategy [9]. Valuable *S*-gene alleles mediating a high level of resistance with a minimum of associated fitness costs are expected to be rare. However, state-of-the-art mutagenesis techniques [19]–[23] allow the creation and or detection of such rare alleles once *S*-genes have been identified in a particular crop species. Here we have shown that the *S*-gene functions of *DMR1* and *PMR4* are conserved between Arabidopsis and tomato, and therefore expected to be functional in other plant species as well. Silencing of *SlPMR4* did not result in severe fitness costs (reduced growth) suggesting that loss-of-function or hypomorphic alleles could mediate powdery mildew resistance with a minimum of fitness costs in tomato. *SlDMR1* silencing was strongly associated with severe fitness costs in tomato and sldmr1 alleles conferring a high level of resistance in absence of severe effects on growth (and yield) might therefore be more difficult to obtain. However, the existence of the Arabidopsis *dmr1-3* mutant that is resistant to downy mildew [7] without severe fitness costs (Figure 3H) suggests that similar alleles can be identified in at least some plant species. Identification of new *S*-genes in model and crop species will contribute to future disease resistance breeding.

For many crop species genome sequences are or will soon become available, which will facilitate the identification of orthologs of *S*-genes in other plant species. Assuming that their function in disease susceptibility is conserved among different plant species, as is the case for MLO [11]–[14], PMR4 and DMR1, desired mutations can easily be obtained by (targeted) mutagenesis approaches [19]–[23] and applied in breeding crops with durable resistance. Thus this study presents a proof-of-concept research on the potential exploitation of orthologs of Arabidopsis *S*-genes in resistance breeding in crop species.

## Supporting Information

File S1 Sequences of silencing fragments used to specifically silence tomato genes *Solyc07g053980* (*SlPMR4*) and *Solyc04g008760* (*SlDMR1*), and experimental data showing the absence of cross-silencing of another tomato PMR4 family member *Solyc02g078230* (*SlPMR4_h2*) by the *SlPMR4_h1* silencing construct.
(DOCX)
