# Supplementary material for: Powdery Mildew Resistance in Tomato by Impairment of SlPMR4 and SlDMR1
Source: PLoS One. 2013 Jun 20;8(6):e67467. doi: 10.1371/journal.pone.0067467 (PMC3688610; doi:10.1371/journal.pone.0067467)
Supplement: File S1 — Sequences of silencing fragments used to specifically silence tomato genes Solyc07g053980 (SlPMR4) and Solyc04g008760 (SlDMR1), and experimental data showing the absence of cross-silencing of another tomato PMR4 family member Solyc02g078230 (SlPMR4_h2) by the SlPMR4_h1 silencing construct. (DOCX) [file pone.0067467.s001.docx]

**Figure S1. Sequence used in silencing constructs**

Sequence of silencing fragment in pUC57 for SlPMR4_h1 (*Solyc07g053980*) AAATAATGATTTTATTTTGACTGATAGTGACCTGTTCGTTGCAACAAATTGATGAGCAATGCTTTTTTATAATGCCAACTTTGTACAAAAAAGCAG

ATGAGCCTCCGGCAACGTTCAACGCCGGCGGCGAGACAAGTTTCTATAGATGAAGAACCATATAACATCATTCCGATTCATAATCTTCTAGCTGACC

CAGCTTTCTTGTACAAAGTTGGCATTATAAGAAAGCATTGCTTATCAATTTGTTGCAACGAACAGGTCACTATCAGTCAAAATAAAATCATTATTT

Sequence of silencing fragment in pUC57 for SlDMR1(*Solyc04g008760*)

AAATAATGATTTTATTTTGACTGATAGTGACCTGTTCGTTGCAACAAATTGATGAGCAATGCTTTTTTATAATGCCAACTTTGTACAAAAAAGCAG

TCGATGGATCAATCAATCAATCAACTATGCCTCAATTCCAAACGAGTTTGGTATTAGTTGTATGAATCCTATATATATCTTTTCACTATATGAATTCATCATGCTATGTGAAAAATGATATGTAGGAATTTGTGTGCAAT

CAGCTTTCTTGTACAAAGTTGGCATTATAAGAAAGCATTGCTTATCAATTTGTTGCAACGAACAGGTCACTATCAGTCAAAATAAAATCATTATTT

In black the attL sites are shown; in blue the silencing fragment.

**Table S1.** Primers used in qPCR analysis to investigate cross-silencing*.

| Primer combination | Forward primer | Reverse primer |
| --- | --- | --- |
| SlPMR4_h1 | GCCGGCGGCGAGACAAGTTT | CAGCGCCAGCCAGTCAAGCA |
| SlPMR4_h2I | TGGCACTGCCCCTCATTCAGC | GCGGCCATTTCAGCCTCCGA |
| SlPMR4_h2II | GGGGCAACGTGTTCTTGCCGA | TGGAAGCCTTGCTGATGCCACC |
| EF-1α | ATTGGAAACGGATATGCCCCT | TCCTTACCTGAACGCCTGTCA |

*For analysis of cross-silencing of SlPMR4_h2 (*Solyc02g078230*) by the SlPMR4_h1 (*Solyc07g053980*) construct qPCR was performed with 2 primer pairs specific for SlPMR4_h2, besides the primer pair specific for SlPMR4_h1. As a reference gene *EF-1α* was chosen.


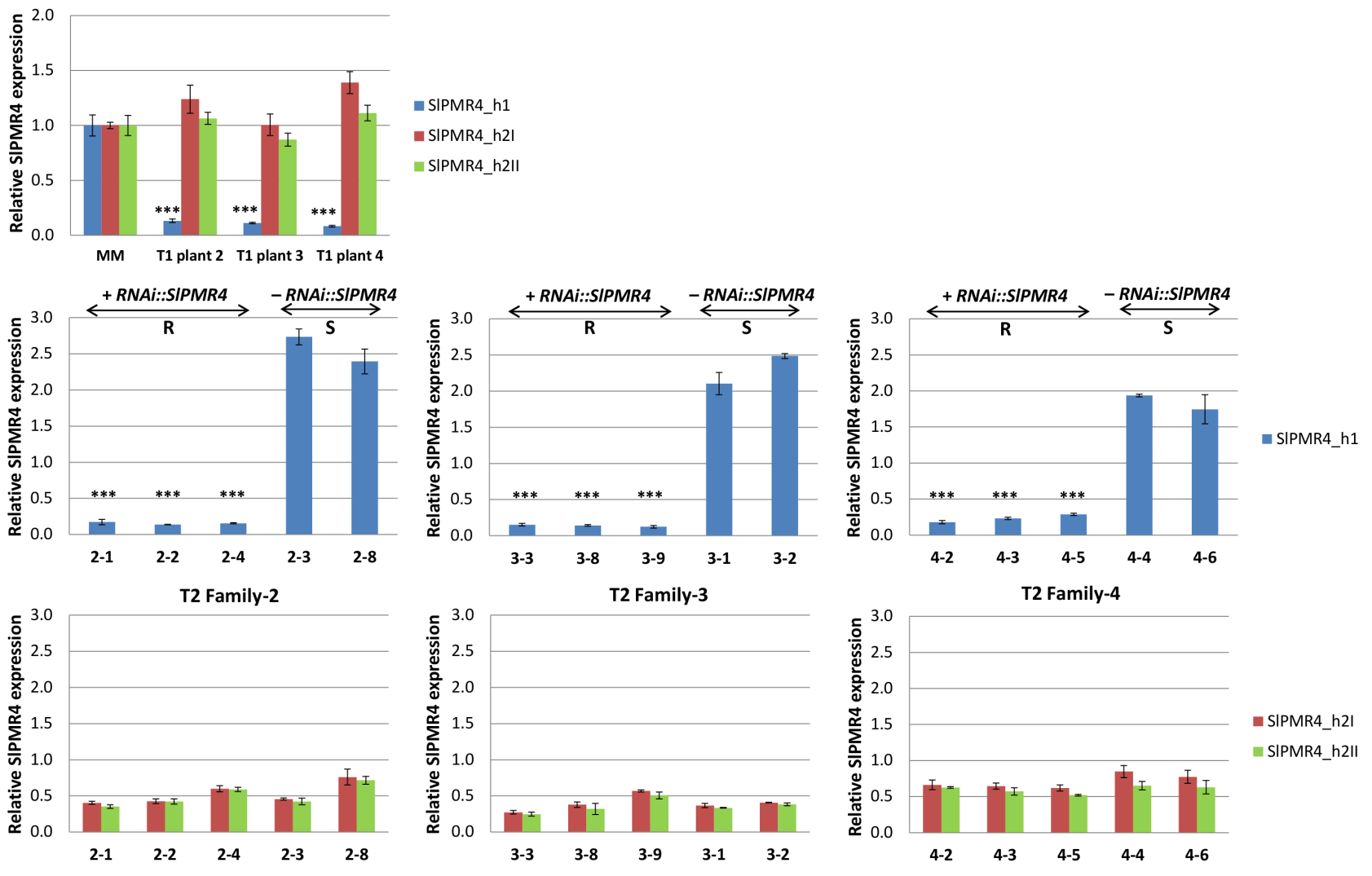


**B**

**A**

**Figure S2. No cross-silencing of SlPMR4_h2 occurs with the silencing construct designed to reduce expression level of SlPMR4_h1.** (**A**) Relative *SlPMR4_h1* and *SlPMR4_h2* expression levels were determined by qPCR in untransformed Moneymaker (MM) plants and three selected T1 transformants that showed a high level of silencing of *SlPMR4_h1*. In these T1 plants expression of SlPMR4_h2 was not significantly changed. (**B**)T2 progeny obtained after selfing the three T1 plants showed segregation of powdery mildew resistant and susceptible plants. Progeny containing the T-DNA sequence showed strongly reduced expression of *SlPMR4_h1* and were resistant. Progeny without T-DNA sequence showed no reduction of expression of *SlPMR4_h1* and were susceptible. All T2 progeny showed similar levels of expression of *SlPMR4_h2*, indicating no cross-silencing had occurred, and powdery mildew resistance was the result of silencing *SlPMR4_h1*.
